# Postpartum Malarial Fever

**Published:** 1883-09

**Authors:** P. D. Burdick


### ARTICLE V.

### POSTPARTUM MALARIAL FEVER. By P. D. BURDICK, M.D.

In this zymotic country, where it would appear at times as though scarcely any animal life is free from their noxious influence, where can be traced evidences of them in nearly every species of disease peculiar to the climate, many disorders deriving their first cause from the contaminating influence which they are supposed to exert, it could scarcely be deemed presumption in one if he were to pause in his wonderment at their universality and the part which they are presumed to play in the etiology of disease, and ponder if, indeed, they are not the prime factor in all the ills which flesh is heir to.

Vague and unsatisfactory as our understanding of miasm is, the term malaria, dedicated to express a condition easier conceived than demonstrated, one that, had not the orthodox definition of that spring in the human breast, described as the substance of things hoped for, the evidence of things unseen, been so long associated with the word used for its appellation, it might be misjudged which it was intended to define, faith or malaria.

To question its nomenclature is to call into question the existence of the hypothetical germ itself, which, at the present stage of our knowledge, one might as well doubt his catechism. It would but expose the "doubting Thomas" to well trained batteries filled to repletion, or place him among the unenviable list of nihilists or heretics, taught in the amphitheater and laboratory that the baneful effects of a fog are due not to the fog itself, but to an impalpable molecule of decomposed vegetable matter, so disguised and tucked away within the little humid sphere as to be beyond the ken of the chemist.

Though analytical chemistry can draw a line so fine among particles of matter as to determine where the atom leaves off and where the molecule begins, and teaches us that the smallest division of matter that can exist in a free state is a molecule; that the surrounding media, termed atmosphere, is made up of oxygen, hydrogen, nitrogen, carbonic acid gas, impurities, etc., giving the exact proportion of each, yet fails to take the fog, which can be caught in a hat, or collects itself all over every thing exposed to its misty sheet, and explain to us what this miasm is that we hear so much of floating around in its vehicle of moisture and what, indeed, are its generic forces.

Professor Salisbury displayed a commendable disposition in catching some vapory emanations upon some fragments of glass and analyzing them, establishing the fact, seemingly to his own satisfaction, that the form of fever generally denominated malarial is the result of the introduction into the system of spores or cells of certain species of algoid plants, termed palmellæ; but it has been a long time since Professor Salisbury and his pieces of glass were heard from, and the impression pretty generally prevails that the only well authenticated point which the professor succeeded in thoroughly establishing is the fact that he was a little too previous.

It is a custom fairly observed when discussing medical topics, and reference is made to the ancients, to do so with a degree of charity, and it is not our plan to break in upon this time-honored custom of the faculty, or to infringe upon any of the established precepts nor trample upon well regulated rules.

When the subject of malaria is thrust upon us our thoughts naturally wander back to that period when, to use the parlance of to-day, the indications of the barometer would have signaled a lowering temperature, and at such times there might have been seen creeping up and down the banks of the inner seas among the bayous and fenlands of that oriental country, wherever the low lands and capricious ill winds favored, bearing with it seeds of fever, which in those days meant depopulation and death, a dense heavy mist or fog.

And when I contemplate on this fevered element a responsive chord in my sympathetic nature is touched, for that people who were so unfortunate as to have existed at a period when the best science of the times attributed a plague which should follow in the wake of a prevailing mist, to the wrath of an offended God ekeing his vengeance out upon them, because of their sins, either of omission or commission, and what is indeed sad, that they should have passed away—gone down to their graves—without ever having heard of the zymotic theory, without ever having learned that their revengeful God wasn't anything but a fairy-like tale of zymots in a fog.

How pleasant would have been the task had it fallen upon, or had it been within our province to acquaint them of all this, providing their natures were not so inquisitive as to insist upon knowing just exactly where the zymotic part of the fog came in.

But I had purposed speaking briefly of a fever which has been producing more or less havoc down in our part of the country; a fever which for convenience sake I shall pass under the *nom-de-plume* of postpartum malarial.

It has been my experience, if the parturient woman is in any way suffering from sluggish secretory organs; if the hepatic circulation is impeded in consequence of engorgement, which I find to be particularly common among females during gestation in this climate, and as a result impaired secretion, indigestion and assimilation; if this condition of things be not critically looked after before and up to the time of labor, the exertion, overwork and shock, which the patient from the very nature of things sustains from the parturient act is very apt to result in what in my judgment is the sequel of it all, a fever.

The immediate danger of which lies not so much in the fever itself if properly handled, as upon an error in diagnosis, which one is very prone to make, owing more particularly to the character which the lochia assumes upon the incipiency of the pyrexia, leading one to believe that the abnormal increase of temperature is wholly dependent upon, and the result of, the absorption into the system, through the exposed vessels of the uterus, of putrescent matter or lochia seriously altered in character.

If this idea is allowed to prevail, and medicines adopted calculated to meet seemingly the marked indications ascribing the constitutional disturbance to purely a local cause, there is danger ahead.

I am misinformed if there have not been some twenty lost in a little city where I had been summoned not long since to see a lady suffering with this malady, which has come to be looked upon by the women of that section as a scourge, dreading the approach of labor with the horror almost of death.

Clinical history discloses the following :

They may feel comparatively well, and it is not at all uncommon for them to have enjoyed good health up to the time labor sets in ; but as a rule they suffer occasionally from slight attacks of biliousness previous to this, generally, in their belief, too slight to justify consulting a physician.

Two, three, or perhaps more days even may elapse after labor —which, supposing it to have been propitious, is over—before any untoward symptoms become manifest. Up to this time, I repeat, in substance, there may not have been a single symptom, or but a single symptom which would cause the accoucheur to anticipate any change in the condition of his patient, or that could possibly lead him to suspect any unfavorable turn in the case, and it is only from my own experience that I can speak in regard to this, and that is the appearance which the tongue presents, which may be said to have to be seen in order to be fully appreciated, for its general characteristics are subject to modifications altogether owing to the temperament of the patient, hence no one feature is invariably present, but as a rule a dark viscid coat is noticeable on its posterior aspect, and this may or may not be accompanied with a slight sallow, tawny, or dirty hue of the skin.

Rigors announce a sudden change in the history of the case. The countenance takes on a pinched and anxious look, in time becoming wearied, as though something appalling was about to come to pass.

The lochia, from this time on, either ceases altogether or becomes scanty and green ; more or less tenderness supervenes over the womb and its appendages, in some cases becoming so severe as to necessitate the adoption of active, energetic measures intended to relieve the pain, at other times so slight as not to give rise to any particular inconvenience.

The pulse rises rapidly, assuming a wiry, vibrating movement, the thermal register indicating great increase of temperature. In fact, there is every evidence of septicæmia, and so rapidly are the symptoms incident to this condition developed, that unless the physician has taken cognizance of the real condition of things, or should he fail to immediately after the decided change takes place, the case receives only such treatment as is calculated to meet the indications of the already alarming symptoms.

The treatment generally adopted for blood poisoning is instituted, antiperiodics in heroic doses, and the overloaded secretions are lost sight of for antiseptics and diffusible stimulants. Cardiac sedatives are used sparingly, sinking spells occur and recur, fear takes possession of the patient, and generally within from two to four days, with varying symptoms, it becomes painfully apparent that her forebodings are only too well grounded, and not the product of a perturbed mind or the hallucinations of a fevered brain, as evidenced by the flagging pulse, the failing strength.

A few more sinking spells, stupor supervenes, the curtain falls, the scene closes, and death claims its victim.

---

THE masterly article written by Dr. E. W. Jenks, and published in the Gynaecological Transactions, on "The Practice of Gynaecology in Ancient Times" has been translated into German, and appears in a recent number of the German "*Archives of the History of Medicine.*"

